# Supplementary material for: Dissipation Behavior and Acute Dietary Risk Assessment of Thiamethoxam and Its Metabolite Clothianidin on Spinach
Source: Molecules. 2022 Mar 29;27(7):2209. doi: 10.3390/molecules27072209 (PMC9000691; doi:10.3390/molecules27072209)
Supplement: Supplementary file 1 [file molecules-27-02209-s001.zip › molecules-1595629-supplementary.pdf]

Supplementary Information

Dissipation behavior and acute dietary risk assessment of thiamethoxam and its metabolite clothianidin on spinach

Table S1. Calibration curves, correlation coefficient (R^2) and matrix effect of thiamethoxam and clothianidin in spinach.

Compound	Matrix	Linear range (μg kg^{-1})	Calibration curves	R^2	ME(%)
Thiamethoxam	Solvent	5-200	y=1436001x+10805.7	0.9951	-
	Spinach	5-200	y=1050908x+4933.6	0.9975	-27
Clothianidin	Solvent	5-200	y=946511x+1251.5	1.0000	-
	Spinach	5-200	y=449745x+106.0	1.0000	-52

Table S2. Residues of thiamethoxam and clothianidin on spinach at different sampling time in Shanxi, Anhui, Guangdong, and Shandong sites.

Analyte	Time /day	Residues (mg kg^{-1})			
		Shanxi	Anhui	Guangdong	Shandong
Thiamethoxam	0	1.2	0.79	0.74	0.35
		1.3	0.83	0.84	0.36
	3	0.15	0.094	0.11	＜0.010
		0.69	0.12	0.17	0.011
	5	0.36	0.040	0.029	＜0.010
		0.41	0.041	0.041	＜0.010
	7	0.10	0.013	0.017	＜0.010
		0.13	0.021	0.018	＜0.010
	10	＜0.010	＜0.010	＜0.010	＜0.010
		0.014	＜0.010	＜0.010	＜0.010
Clothianidin	0	0.073	0.11	0.25	0.50
		0.110	0.14	0.32	0.52
	3	0.20	0.23	0.43	0.16
		0.45	0.26	0.55	0.17

	5	0.42	0.20	0.43	0.067
		0.44	0.21	0.52	0.068
	7	0.32	0.14	0.34	0.030
		0.32	0.19	0.33	0.034
	10	0.059	0.037	0.24	0.014
		0.092	0.078	0.30	0.016

Table S3. Experiment information and climate types of the field sites.

No.	Location	Cultivation facilities	Climate type
1	Inner Mongolia	Greenhouse	Temperate continental monsoon climate
2	Shanxi	Greenhouse	Temperate continental monsoon climate
3	Beijing	Greenhouse	Warm temperate and semi humid continental monsoon climate
4	Shandong	Open field	Temperate monsoon climate
5	Anhui	Open field	Temperate monsoon climate
6	Hunan	Open field	Subtropical monsoon climate
7	Guizhou	Open field	Subtropical humid monsoon climate
8	Guangdong	Shade mesh	Subtropical monsoon climate

Table S4. Detailed information about the application process.

Location	Calibration/ Application Date	Mean Spray velocity (mL/s)	Area of Treated Plots	Formulated Product (mL)	Carrier (Water) (L)	Volume of solution (L)	
						left	Applied
Inner Mongolia	6/5/2020	14.60	50	112.5	2.25	0	2.25
Shanxi	8/31/2020	13.30	50	112.5	2.25	0	2.25
Beijing	6/11/2020	30.36	50	112.5	2.25	0	2.25
Shandong	7/7/2020	14.00	50	135.0	2.70	0.49	2.21
Anhui	10/11/2020	19.40	50	135.0	3.60	0.52	3.08
Hunan	11/5/2020	23.13	50	112.5	4.50	0.21	4.26
Guizhou	10/23/2020	15.73	50	135.0	3.60	0.48	3.12
Guangdong	11/16/2020	15.30	56	160.0	3.20	0.56	2.64

Table S5. Instrument parameters of thiamethoxam and clothianidin in multiple reaction monitoring (MRM) mode.

Column Temp (°C):	40			
Sample tray (°C):	20			
Injection Volume (μL):	5			
Conditions:	T/min	%A	%B	Flow
	0.00	90	10	0.30
	1.00	90	10	0.30
	1.10	10	90	0.30
	3.00	10	90	0.30
	3.10	90	10	0.30
	5.00	90	10	0.30
MS System:				
Analyte Monitored	Parent ion (m/z)	Daughter ion (m/z)	Cone (V)	Collision (eV)
Thiamethoxam	291.9	210.9*/132.0	30	10/22
Clothianidin	249.9	168.9*/132.0	16	12/12
Ion Source	Electronic Spray Ion			
Source Temp(ºC)	150			
Ion mode	Electrospray+			
Capillary (kV)	3.2			
Desolvation Temp (ºC)	350			
Desolvation（L/Hr）	650			
Collision Gas	Ar,>99.999%			

*Transition for quantification

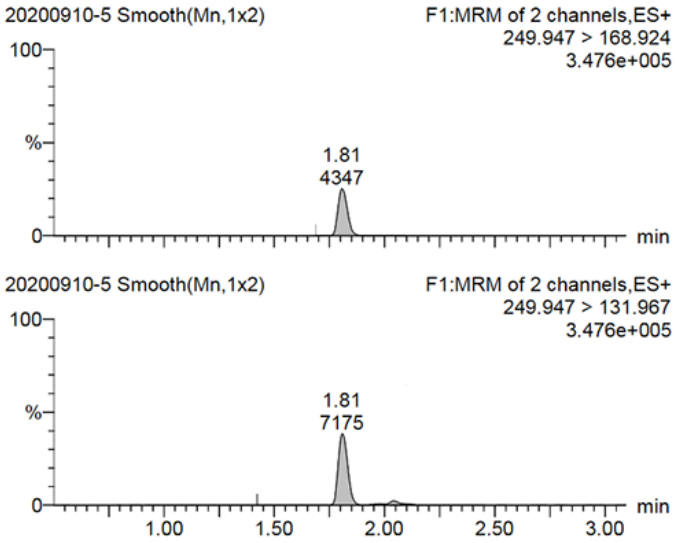

Figure S1. Representative chromatograms (MRM mode) of thiamethoxam in spinach (0.02 mg L^{-1}).

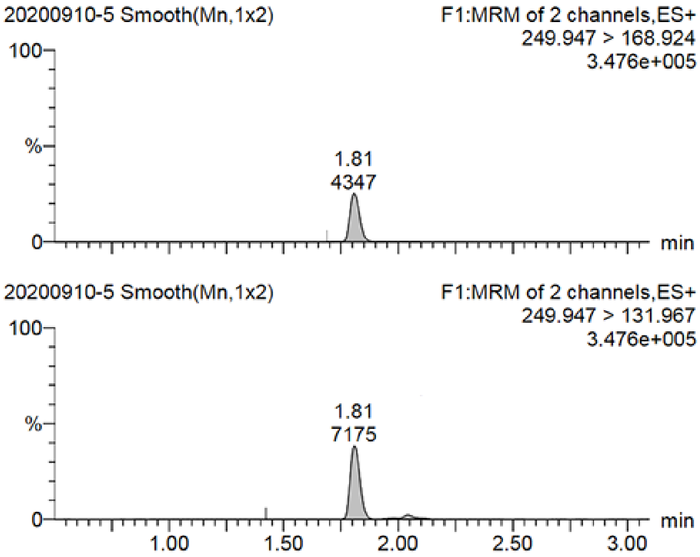

Figure S2. Representative chromatograms (MRM mode) of clothianidin in spinach (0.02 mg L^{-1}).

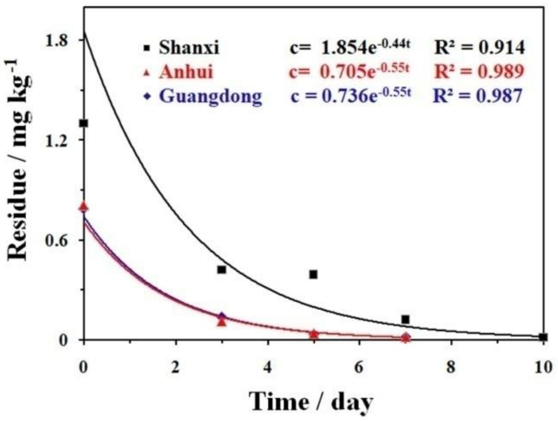

Figure S3. Dissipation kinetics of thiamethoxam fitted with first-order kinetic equation.
